# Effect of Faricimab versus Aflibercept on Hyperreflective Foci in Patients with Diabetic Macular Edema from the YOSEMITE/RHINE Trials

**DOI:** 10.1016/j.xops.2025.100798

**Published:** 2025-04-19

**Authors:** Usha Chakravarthy, Varun Chaudhary, Srinivas R. Sadda, Colin S. Tan, Stela Vujosevic, Sascha Fauser, Kara Gibson, Carl Glittenberg, Nancy Holekamp, Ben Lanza, Andreas Maunz, Jeffrey R. Willis, Rishi P. Singh

**Affiliations:** 1 Centre for Public Health, Queen's University of Belfast, Belfast, UK; 2 Department of Surgery, Hamilton Regional Eye Institute, McMaster University, Hamilton, Ontario, Canada; 3 Doheny Eye Institute, Pasadena, California; 4 Department of Ophthalmology, David Geffen School of Medicine, University of California, Los Angeles, California; 5 National Healthcare Group Eye Institute, Tan Tock Seng Hospital, Singapore; 6 Department of Biomedical Surgical and Dental Sciences, University of Milan, Milan, Italy; 7 Eye Clinic, IRCCS MultiMedica, Milan, Italy; 8 F. Hoffmann-La Roche Ltd., Basel, Switzerland; 9 Roche Products Ltd., Welwyn Garden City, UK; 10 Pepose Vision Institute, Chesterfield, Missouri; 11 Genentech, Inc., South San Francisco, California; 12 Center for Ophthalmic Bioinformatics, Cole Eye Institute, Cleveland Clinic, Cleveland, Ohio

**Keywords:** Angiopoietin-2, Diabetic macular edema, Faricimab, Hyperreflective foci, VEGF

## Abstract

**Purpose:**

To compare the effect of faricimab, a dual angiopoietin-2 (Ang-2) and VEGF-A inhibitor, with aflibercept on resolution of hyperreflective foci (HRF) in patients with diabetic macular edema (DME).

**Design:**

A post hoc analysis of the randomized, double-masked, noninferiority YOSEMITE/RHINE (NCT03622580/NCT03622593) phase III trials.

**Participants:**

Adults with vision loss due to center-involving DME.

**Methods:**

A deep learning–based algorithm was used to automatically quantify HRF in spectral-domain OCT volume scans from YOSEMITE/RHINE. Study eyes were randomized to faricimab 6.0 mg every 8 weeks (Q8W; n = 519), faricimab 6.0 mg according to a personalized treat-and-extend (T&E)–based regimen (n = 524), and aflibercept 2.0 mg Q8W (n = 502). Hyperreflective foci were defined as hyperreflective objects up to 50 μm in diameter and assessed within the 1.0-mm and 3.0-mm–diameter ETDRS rings and by location within the inner and outer retina.

**Main Outcome Measures:**

Hyperreflective foci volume and count at baseline and over time through week 48 in the inner, outer, and total retina, 1-mm and 3-mm diameters; time to absence of HRF at 2 consecutive visits in the inner and outer retina, 1-mm diameter over 48 weeks.

**Results:**

Adjusted mean HRF volumes at week 48 were lower for faricimab Q8W (104.1 picoliter [pL]) and faricimab T&E (110.1 pL) compared with aflibercept (180.3 pL; nominal *P* < 0.001 for both) in the inner retina, 1-mm diameter. In the inner retina, 3-mm diameter adjusted mean HRF volumes at week 48 were lower for faricimab Q8W (763.9 pL) and faricimab T&E (777.2 pL) compared with aflibercept (1030.6 pL; nominal *P* < 0.001 for both). Similar results were obtained for volumes in the outer retina and for HRF counts. In the inner retina, 1-mm diameter, the 25th percentile for time to absence of HRF count at 2 consecutive visits was achieved 8 weeks earlier with faricimab Q8W and faricimab T&E versus aflibercept.

**Conclusions:**

Greater HRF reductions were achieved with faricimab versus aflibercept, supporting the therapeutic potential of dual Ang-2/VEGF-A inhibition to suppress disease activity in DME.

**Financial Disclosure(s):**

Proprietary or commercial disclosure may be found in the Footnotes and Disclosures at the end of this article.

Hyperreflective foci (HRF) have been proposed as biomarkers of clinical significance linked to disease severity and treatment response in diabetic macular edema (DME).^1^, ^2^, ^3^, ^4^ Hyperreflective foci are small, distinct objects that generate a highly reflective signal on spectral-domain OCT (SD-OCT).^5^ The origin of HRF in DME remains under investigation, and there are 2 main hypotheses on the morphological correlates of HRF detected by SD-OCT. The first proposes that HRF are activated microglia or infiltrated leukocytes, thus representing a retinal inflammatory response.^6^, ^7^, ^8^, ^9^ The second hypothesis suggests that HRF are protein and/or lipid exudates resulting from the breakdown of the blood–retinal barrier.^5^^,^^10^, ^11^, ^12^ As these hypotheses are not mutually exclusive, some groups have also discussed the possibility of both hypotheses applying concurrently, with smaller HRF (≤30 μm) proposed to correspond to inflammatory cells and larger objects (>30 μm) representing macromolecular exudates.^3^^,^^13^ Histological studies on HRF are currently lacking in DME and are necessary to confirm these hypotheses. A better understanding of the origin of HRF will help elucidate the role of HRF in DME, including response to treatment.

Faricimab is the first bispecific antibody designed for intravitreal injection that independently binds to and neutralizes angiopoietin-2 (Ang-2) and VEGF-A. Preclinical studies have shown that Ang-2 and VEGF-A can act in synergy to drive vascular leakage, neovascularization, and inflammation, supporting combined inhibition of Ang-2 and VEGF-A as a potentially valuable approach to promote vascular stability and improve long-term outcomes in DME.^14^, ^15^, ^16^, ^17^, ^18^

The phase III YOSEMITE and RHINE trials evaluated intravitreal faricimab in patients with DME.^19^, ^20^, ^21^ Compared with aflibercept every 8 weeks (Q8W), faricimab Q8W and faricimab treat-and-extend (T&E) demonstrated comparable visual acuity gains and better and more sustained anatomic outcomes, including greater central subfield thickness (CST) reductions and more patients achieving absence of DME that were maintained through year 2.^20^^,^^21^ These trials demonstrated that dual inhibition of VEGF-A and Ang-2 leads to robust functional and anatomic outcomes with the potential to reduce treatment burden. The dual Ang-2/VEGF-A inhibition offered by faricimab may additionally have the potential to abrogate the ongoing inflammatory components that drive DME.

To help understand the role of HRF as a biomarker in DME, we recently developed a deep learning–based algorithm with a robust performance for automated assessment of HRF in DME.^22^ Using this algorithm, HRF were automatically segmented in SD-OCT volume scans from the YOSEMITE and RHINE trials at baseline. A treatment-agnostic analysis showed that HRF were almost universally present in eyes with DME and were linked to disease severity at baseline. Furthermore, the spatial distribution of HRF in the en face projection closely followed that of cystoid intraretinal fluid (IRF) at baseline.^22^

The aim of this exploratory post hoc analysis of YOSEMITE and RHINE was to assess the impact of faricimab compared with aflibercept on HRF resolution over 48 weeks in patients with DME using an automated deep learning–based algorithm.

## Methods

### Study Design

The study design and rationale for YOSEMITE (NCT03622580) and RHINE (NCT03622593) have been described previously.^19^, ^20^, ^21^ In brief, YOSEMITE and RHINE were identical, randomized, double-masked, noninferiority, phase III clinical trials that assessed the efficacy, durability, and safety of faricimab in anti-VEGF treatment-naïve and previously treated (limited to 25%) adults (aged ≥18 years) with vision loss due to center-involving DME (CST ≥325 μm; best-corrected visual acuity [BCVA] of 25–73 ETDRS letters).^19^, ^20^, ^21^ Eligible patients were randomized 1:1:1 to intravitreal faricimab 6.0 mg Q8W after 6 initial every-4-week (Q4W) doses, intravitreal faricimab 6.0 mg T&E with up to 16-week intervals after 4 initial Q4W doses, or intravitreal aflibercept 2.0 mg Q8W after 5 initial Q4W doses, up to week 96. To maintain masking, all patients were monitored Q4W and received sham injections at nonactive dosing visits. The final study visit was at week 100.

YOSEMITE and RHINE were conducted in accordance with the International Council for Harmonization E6 Guideline for Good Clinical Practice, tenets of the Declaration of Helsinki, US Food and Drug Administration regulations, and European Union Clinical Trials Directive (2001/20/EC) as appropriate; and all applicable local, state, and federal laws. Institutional review board/ethics committee approval was obtained for study protocols before trial commencement. All patients provided written informed consent to participate.

### Data Selection

For the current post hoc analysis, data up to week 48 were pooled from the YOSEMITE and RHINE trials. All available study eyes with Spectralis (Heidelberg Engineering) volume scans (97 B-scans; scan spacing of ∼62 μm) at any timepoint were included in the analysis population (N = 1545), providing a set of 519, 524, and 502 patients treated with faricimab 6.0 mg Q8W, faricimab 6.0 mg T&E, and aflibercept 2.0 mg Q8W, respectively. The distribution of observed baseline scan areas was 32.2 mm^2^, 34.5 mm^2^, and 37.4 mm^2^ for the 10th, 50th, and 90th percentiles, respectively. The analysis set was limited to study eyes with Spectralis volume scans at the baseline visit (N = 1527); all available images from subsequent visits were analyzed. In order to obtain a homogenous data set, volumes acquired on Zeiss (N = 150) and Topcon (N = 3) devices were excluded. Spectral-domain OCT volume scans from the first year (baseline to week 48) were retrieved, and all available volumes with 97 B-scans were segmented with the model described below.

### Assessment of HRF

Hyperreflective foci on SD-OCT were defined as distinct bright dots of similar reflectivity to the retinal pigment epithelium (RPE) that were ≥20 μm to ≤50 μm in diameter. The lower limit was selected based on the resolution limit of SD-OCT. The upper size limit for HRF in DME reported in the literature ranges from 30 to 50 μm.^3^^,^^6^^,^^23^, ^24^, ^25^, ^26^, ^27^, ^28^, ^29^ We selected 50 μm as the upper limit to capture HRF that may be aggregated into hyperreflective objects >30 μm.^22^

The HRF segmentation model has been described previously and received median and average Dice scores of 71% and 65%, respectively, and median and average specificity on the validation set of 76% and 69%, respectively.^22^ In brief, the deep learning–based algorithm was trained with manually annotated B-scans from the phase II BOULEVARD (NCT02699450) DME trial. The graders characterized intraretinal hyperreflective material according to origin as follows: (1) exudate, (2) blood, and (3) other. To ensure that blood vessels were not detected, the segmentation model was trained on the exudative intraretinal hyperreflective material only. Objects detected by the model were categorized on the B-scan level by fitting an ellipse to each object; objects ≤50 μm in diameter (long axis) were classified as HRF, and objects >50 μm in diameter were discarded. Counts of distinct HRF objects and total HRF volume were automatically extracted on the SD-OCT B-scan and SD-OCT volume level, respectively. Volumes were calculated using the depth information from the slice thickness (the space between the centers of 2 adjacent B-scans). Hyperreflective foci counts and volumes were assessed in the 1-mm and 3-mm ETDRS rings and by location within the inner and outer retina. The inner retina was defined as the internal limiting membrane to, and including, the outer plexiform layer-Henle fiber layer. The outer retina was defined from the outer plexiform layer-Henle fiber layer to the RPE. The total retina was defined as the internal limiting membrane to the RPE. Owing to significant variability in scan area for Spectralis volume scans despite identical parameter settings, the 6-mm diameter ring was excluded from the analysis, as it was not always fully covered. For the same reason, volume-wide measurements across the full OCT scan are potentially unreliable because they are not comparable.

### Outcomes

Outcomes of this post hoc analysis included HRF volume and count at baseline and over time through to week 48 in the inner, outer, and total retina; 1-mm and 3-mm diameters; and time to absence of HRF count at 2 consecutive visits in the inner and outer retina, 1-mm diameter over 48 weeks. A correlation analysis was performed to assess the relationship between change in HRF volume and change in IRF volume in the total retina, 3-mm diameter at week 48.

### Statistical Analysis

Mean values and treatment group comparisons were estimated for each HRF parameter separately using a mixed model for repeated measures, adjusted for baseline HRF value, treatment arm, visit, visit-by-treatment-arm interaction, and baseline BCVA, as well as the randomization-stratification factors: baseline BCVA category (<64 vs. ≥ 64 letters); prior intravitreal anti-VEGF therapy (yes or no); and region (United States and Canada, Asia, and rest of the world). For HRF volumes, mixed model for repeated measure analyses were performed on original units (μm^3^) but axis values were converted to picoliter (pL) for mean plots. An unstructured covariance structure was assumed. Given the positively skewed distribution of the data, sensitivity analyses were conducted using a stratified Wilcoxon rank-sum test.

Kaplan–Meier analyses were performed with associated log-rank tests to estimate the probability of achieving the absence of an HRF count at 2 consecutive visits in patients without an HRF count of zero at baseline. Absence of HRF count was defined as the first time of HRF count = 0 for 2 consecutive visits, with the aim to demonstrate that HRF resolution was maintained for >1 visit. Patients were censored if lost to follow-up/dropped out before the occurrence of the event or if they were still in the study at week 48 without having the event. Statistics for pairwise comparisons were calculated using a separate model for each comparison. Hazard ratios were estimated by Cox regression stratified by baseline BCVA (<64 vs. ≥64 letters), prior intravitreal anti-VEGF therapy (yes vs. no), and region (North America, Asia, and the rest of the world).

A correlation analysis between change in HRF volume and change in IRF volume was performed. The *r* and *P* values were calculated using the Pearson correlation coefficient and corresponding 2-sided alternative hypothesis. The Pearson correlation coefficient was calculated between the change from baseline to week 48 in the cube root–transformed HRF volume and the cube root–transformed IRF volume.

No adjustment to the significance level was made to account for multiple treatment comparisons or analyses at multiple time points. *P* values are nominal and should be interpreted in an exploratory context.

## Results

### Baseline Characteristics

Patient and ocular characteristics at baseline were similar between treatment groups (faricimab 6.0 mg Q8W, faricimab 6.0 mg T&E, and aflibercept 2.0 mg Q8W) for patients from YOSEMITE and RHINE included in this data analysis (Table 1). Further, the baseline characteristics were consistent to those of the overall YOSEMITE/RHINE population.^21^

**Table 1:** Baseline Patient Characteristics, HRF Population

Characteristic	Faricimab 6.0 mg Q8W (N = 519)	Faricimab 6.0 mg T&E (N = 524)	Aflibercept 2.0 mg Q8W (N = 502)
Patient demographics			
Age (yrs), mean (SD)	61.9 (9.62)	62.0 (10.11)	62.3 (9.64)
Female, n (%)	204 (39.3)	195 (37.2)	215 (42.8)
Geographic region, n (%)			
Asia	60 (11.6)	60 (11.5)	48 (9.6)
Europe	143 (27.6)	140 (26.7)	137 (27.3)
North America	263 (50.7)	269 (51.3)	264 (52.6)
Oceania	9 (1.7)	8 (1.5)	8 (1.6)
South America	44 (8.5)	47 (9.0)	45 (9.0)
Race, n (%)∗			
American Indian or Alaska Native	6 (1.2)	5 (1.0)	7 (1.4)
Asian	49 (9.4)	52 (9.9)	43 (8.6)
Black or African American	39 (7.5)	44 (8.4)	33 (6.6)
Native Hawaiian or other Pacific Islander	4 (0.8)	0 (0.0)	3 (0.6)
White	398 (76.7)	399 (76.1)	403 (80.3)
Hispanic or Latino, n (%)	85 (16.4)	102 (19.5)	91 (18.1)
Ocular characteristics			
BCVA (ETDRS letters), mean (SD)	62.2 (9.63)	62.3 (9.55)	62.2 (9.33)
CST (μm), mean (SD)	475.3 (126.69)	477.7 (127.37)	484.5 (130.76)
Macular ischemic nonperfusion, n (%)	212 (44.2)	211 (43.5)	213 (46.2)
Macular leakage, n (%)	497 (100.0)	503 (100.0)	476 (99.8)
Time since DME diagnosis (mos), mean (SD)	16.1 (27.88)	19.4 (34.57)	19.4 (33.99)
Previously anti-VEGF treated, n (%)	113 (21.8)	111 (21.2)	112 (22.3)
ETDRS-DRSS status, n (%)			
DR absent/questionable; mild to moderate NPDR (ETDRS-DRSS level 10/12, 14/20, 35, 43)	296 (57.5)	292 (55.7)	278 (56.2)
Moderately severe to severe NPDR (ETDRS-DRSS level 47, 53)	179 (34.8)	168 (32.1)	174 (35.2)
PDR (ETDRS-DRSS level 61, 65, 71)	34 (6.6)	54 (10.3)	33 (6.7)
Cannot grade (ETDRS-DRSS level 90)	6 (1.2)	10 (1.9)	10 (2.0)

BCVA = best-corrected visual acuity; CST = central subfield thickness; DME = diabetic macular edema; DR = diabetic retinopathy; DRSS = Diabetic Retinopathy Severity Scale; HRF = hyperreflective foci; NPDR = nonproliferative diabetic retinopathy; PDR = proliferative diabetic retinopathy; Q8W = every 8 weeks; SD = standard deviation; T&E = treat-and-extend.

∗ Not all race categories are listed; therefore, the sum of proportions shown does not equal 100%.

### HRF Volume and Count from Baseline to Week 48

At baseline, mean and median HRF volumes in the 1-mm diameter were greater in the inner retina compared with the outer retina, particularly for the median HRF volumes, which were approximately twofold greater in the inner retina versus the outer retina (Table S2, available at www.ophthalmologyscience.org). In the 3-mm diameter ring, mean and median volumes of HRF were similar in the inner and outer retina (Table S2). Analysis by treatment arm showed similar values indicating a balanced distribution.

**Figure 1:**
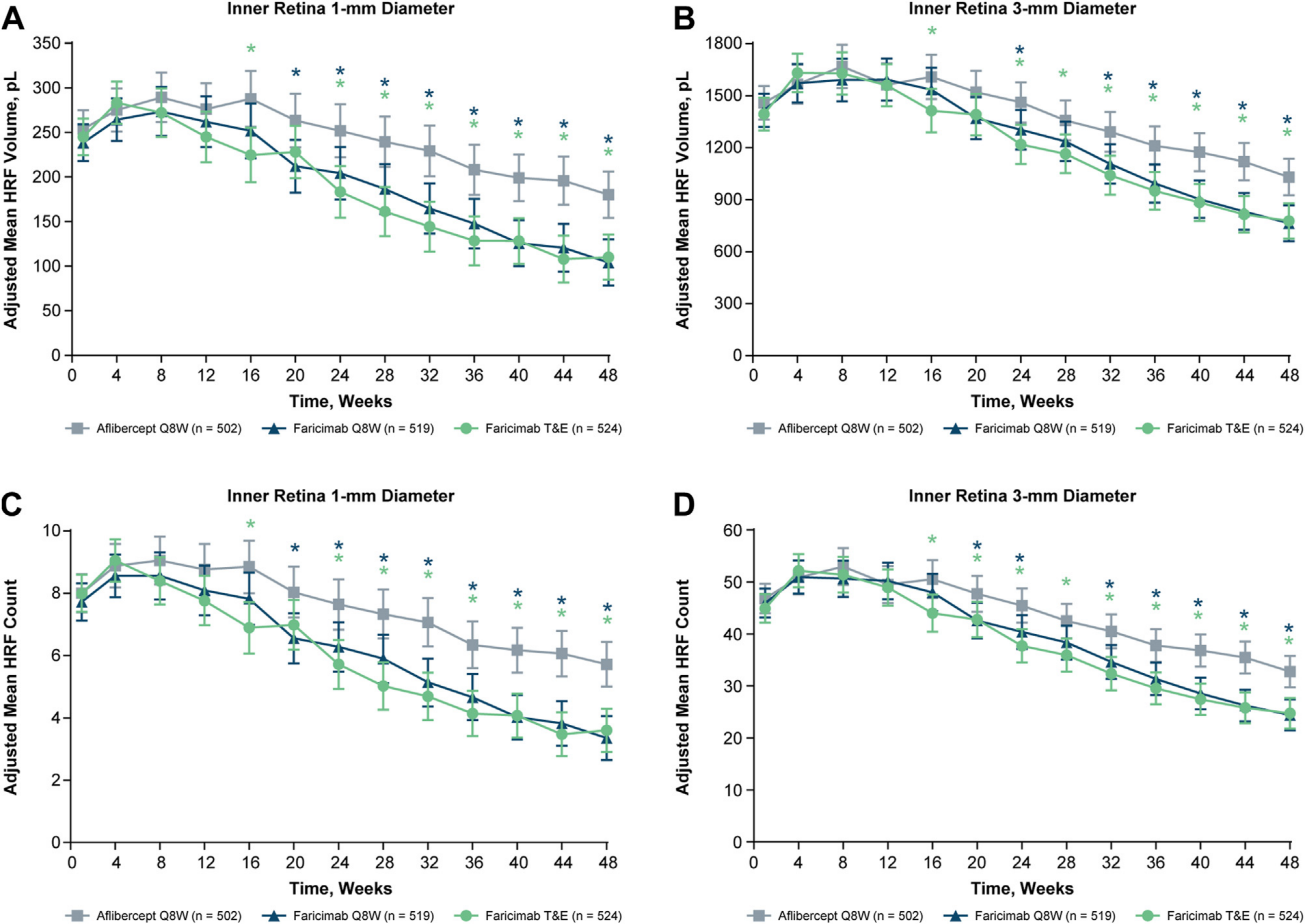
Analysis of HRF volumes and counts in the inner retina over time. Hyperreflective foci volumes in the inner retina **(A)** 1-mm and **(B)** 3-mm–diameter ETDRS rings. Hyperreflective foci counts in the inner retina **(C)** 1-mm and **(D)** 3-mm–diameter ETDRS rings. ∗Nominal *P* < 0.05 vs. aflibercept Q8W. Results and nominal *P* values were obtained using a mixed model for repeated measures analysis. Because the model is adjusted for baseline HRF value, no baseline values are shown in the figure. Error bars are 95% CI. CI = confidence interval; HRF = hyperreflective foci; pL = picoliter; Q8W = every 8 weeks; T&E = treat-and-extend.

**Figure 2:**
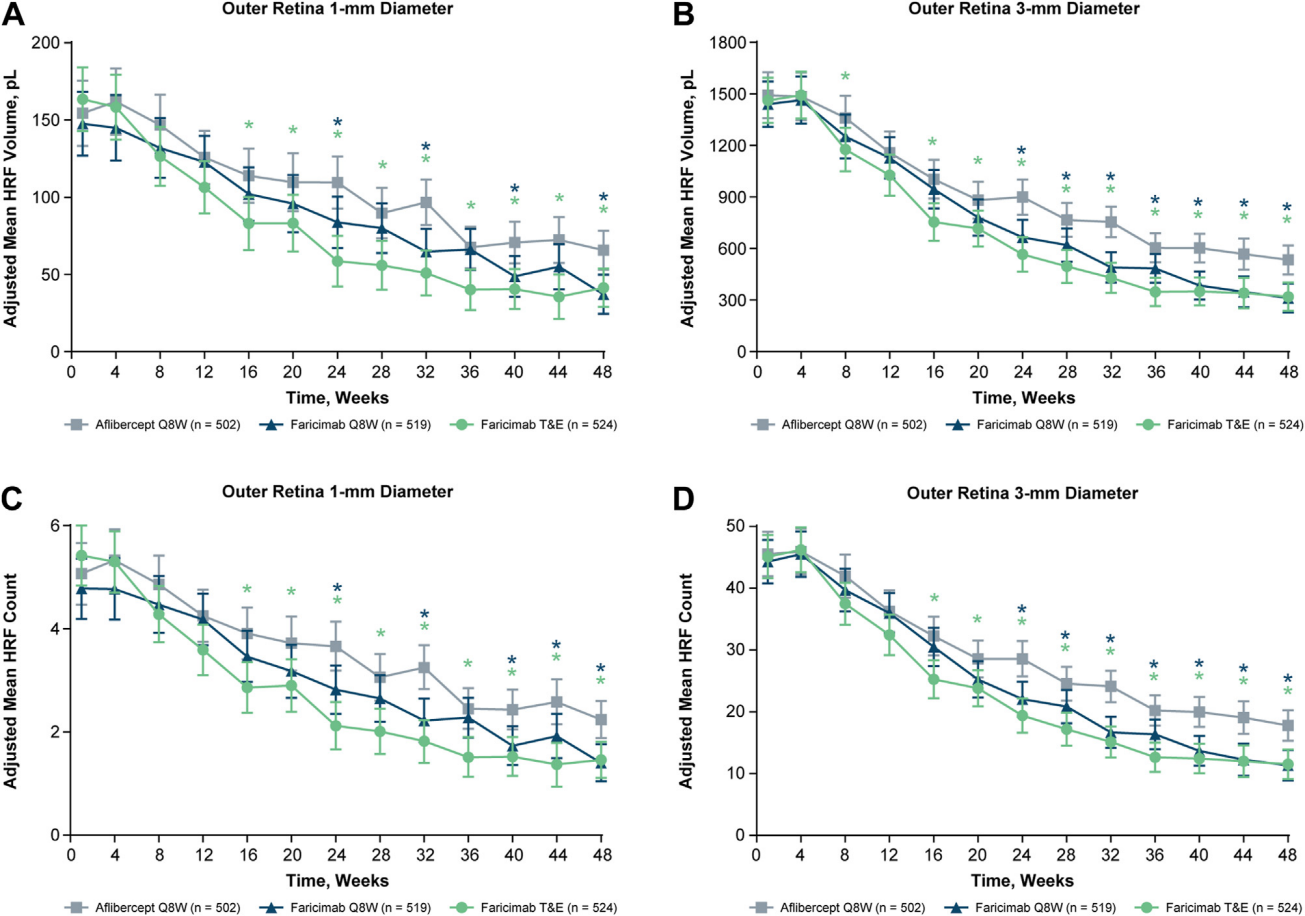
Analysis of HRF volumes and counts in the outer retina for faricimab Q8W (blue), faricimab T&E (green), and aflibercept Q8W (gray) over time. Hyperreflective foci volumes in the outer retina **(A)** 1-mm and **(B)** 3-mm–diameter ETDRS rings. Hyperreflective foci counts in the outer retina **(C)** 1-mm and **(D)** 3-mm–diameter ETDRS rings. ∗Nominal *P* < 0.05 vs. aflibercept Q8W. Results and nominal *P* values were obtained using a mixed model for repeated measures analysis. Because the model is adjusted for baseline HRF value, no baseline values are shown in the figure. Error bars are 95% CI. CI = confidence interval; HRF = hyperreflective foci; pL = picoliter; Q8W = every 8 weeks; T&E = treat-and-extend.

Quantitative assessment over time showed a decrease in HRF volume and count in all treatment arms (Figure 1, Figure 2). In the inner retina after treatment initiation, there was an initial increase in adjusted mean HRF volume for all 3 treatment arms (range, 13%–36%, depending on treatment arm and diameter; Table S3, available at www.ophthalmologyscience.org), peaking at approximately week 8 (Fig 1A, B). The same pattern was seen with the HRF count in the inner retina (Fig 1C, D). Thereafter, HRF volumes and counts decreased steadily over time in the inner retina. There was a more rapid and marked decrease in HRF in the inner retina with faricimab compared with aflibercept Q8W, regardless of regimen (Q8W or T&E). Adjusted mean HRF volumes at week 48 were lower for faricimab Q8W (104.1 pL) and faricimab T&E (110.1 pL) compared with aflibercept (180.3 pL; nominal *P* < 0.001 for both) in the inner retina, 1-mm diameter. Similar results were seen in the inner retina, 3-mm diameter; adjusted mean HRF volumes at week 48 were lower for faricimab Q8W (763.9 pL) and faricimab T&E (777.2 pL) compared with aflibercept (1030.6 pL; nominal *P* < 0.001 for both). This difference was seen from week 16 (the completion of the head-to-head dosing period) onwards (Fig 1 and Table S2).

In contrast to the inner retina, there was no initial increase in HRF volume (Fig 2A, B) or count (Fig 2C, D) in the outer retina. Hyperreflective foci volumes and counts in the outer retina decreased for all 3 treatment arms over time, with greater decreases in the faricimab Q8W and faricimab T&E groups compared with aflibercept Q8W from week 16 onward. In the outer retina, 1-mm diameter adjusted mean HRF volumes at week 48 were lower for faricimab Q8W (37.2 pL) and faricimab T&E (41.4 pL) compared with aflibercept (65.7 pL; nominal *P* < 0.001 and *P* = 0.003, respectively). Similarly, in the outer retina, 3-mm diameter adjusted mean HRF volumes at week 48 were lower for faricimab Q8W (311.7 pL) and faricimab T&E (319.6 pL) compared with aflibercept (533.6 pL; nominal *P* < 0.001 for both). Results were similar for HRF volumes and counts in the total retina (nominal *P* < 0.001 for each faricimab arm compared with aflibercept Q8W at week 48; Fig S3, available at www.ophthalmologyscience.org).

**Figure 4:**
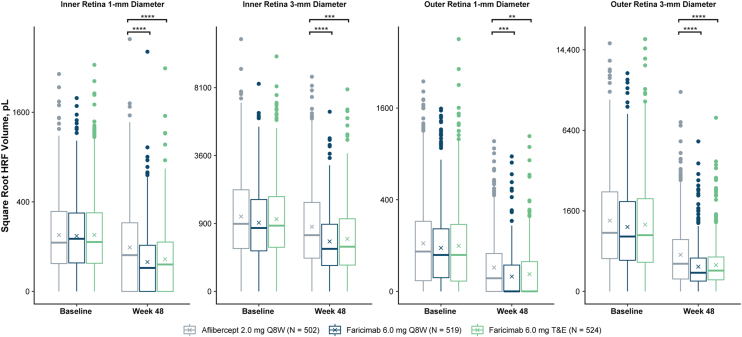
Baseline and week 48 measured HRF volumes in the inner retina 1-mm and 3-mm–diameter and outer retina 1-mm and 3-mm–diameter ETDRS rings for faricimab Q8W (blue), faricimab T&E (green), and aflibercept Q8W (gray). Nominal *P* values, derived from the mixed model for repeated measures, are indicated as ∗∗*P* ≤ 0.01, ∗∗∗*P* ≤ 0.001, and ∗∗∗∗*P* ≤ 0.0001 vs. aflibercept Q8W. Values are square-root–transformed for improved visibility. HRF = hyperreflective foci; pL = picoliter; Q8W = every 8 weeks; T&E = treat-and-extend.

Overall, from baseline to week 48, the decrease in HRF volume with aflibercept Q8W was predominantly in the outer retina, whereas decreases in HRF volume with faricimab Q8W and faricimab T&E occurred in both the inner and outer retina (Fig 4). In the total retina at week 48, the decrease in HRF volume was greater with faricimab Q8W and faricimab T&E compared with aflibercept Q8W (Fig S5, available at www.ophthalmologyscience.org).

**Figure 6:**
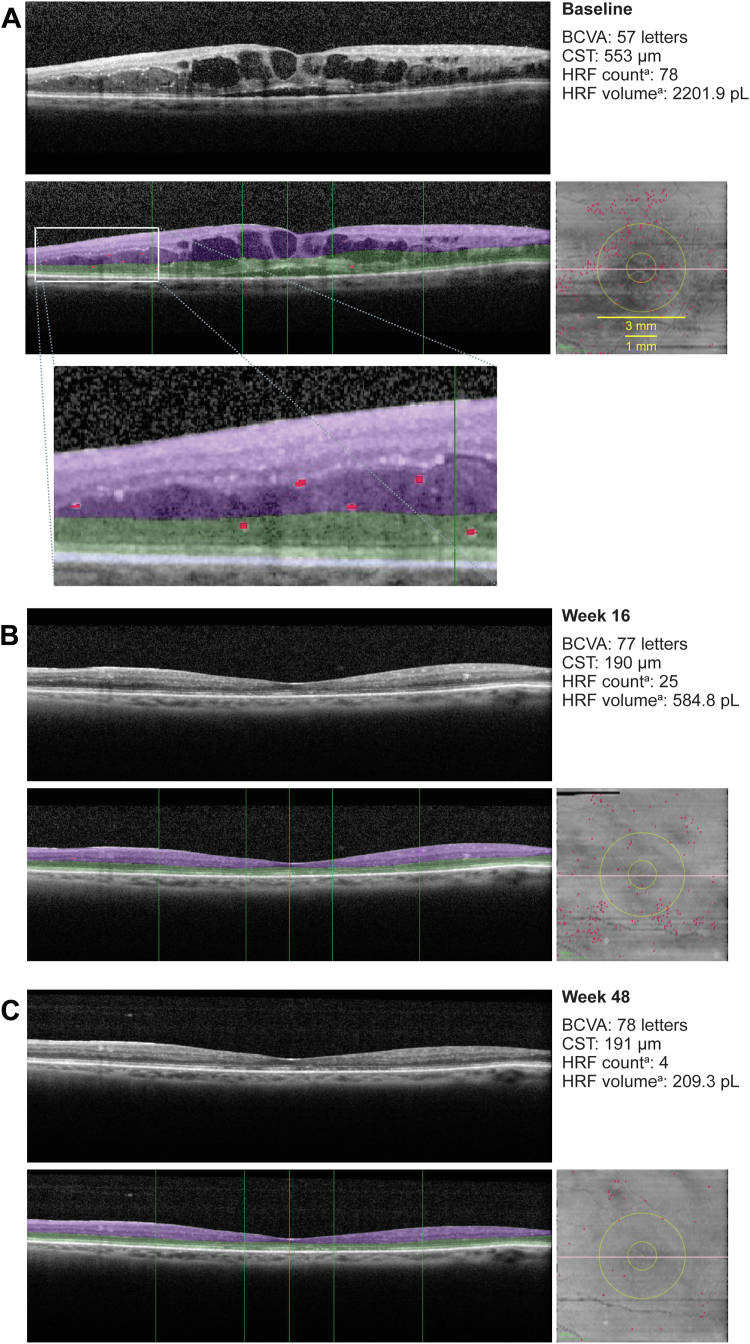
Example of automated HRF segmentation. Spectral-domain OCT images are from 1 patient (77 years of age, female, White, faricimab T&E arm) at **(A)** baseline without (top panel) and with segmentation (bottom panel) of layers; HRF (red; >20 and ≤50 μm); white box with dashed lines indicates left-hand side of image that is enlarged below to focus on segmented HRF. (**B**) Image from the same patient at week 16 without (top panel) and with (bottom panel) segmentation of HRF. (**C**) Image from the same patient at week 48 without (top panel) and with (bottom panel) segmentation of HRF. ETDRS rings are indicated with green vertical lines (center, 1-mm and 3-mm–diameter rings). Inner retina (between the internal limiting membrane and the OPL-HFL; purple) and outer retina (between the OPL-HFL and the retinal pigment epithelium; green). ^a^HRF count and volume are in the total retina 3-mm diameter. BCVA = best-corrected visual acuity; CST = central subfield thickness; HRF = hyperreflective foci; pL = picoliter; OPL-HFL = outer plexiform layer-Henle fiber layer; T&E = treat-and-extend.

Example SD-OCT scans with and without HRF segmentation at baseline, week 16, and week 48 from a patient who received faricimab T&E are shown in Figure 6. Vision and CST were improved at week 16 compared with baseline and were maintained to week 48. Hyperreflective foci count and volume were decreased at week 16 compared with baseline and were further decreased at week 48. A 3-dimensional animation of the same patient at day 1, week 16, and week 48 is shown in Clip S1 (available at www.ophthalmologyscience.org), which highlights the data that are segmented across the entire SD-OCT volume. Fluid volumes were reduced, and HRF was decreased at weeks 16 and 48.

### Time to Absence of HRF Count at 2 Consecutive Visits

**Figure 7:**
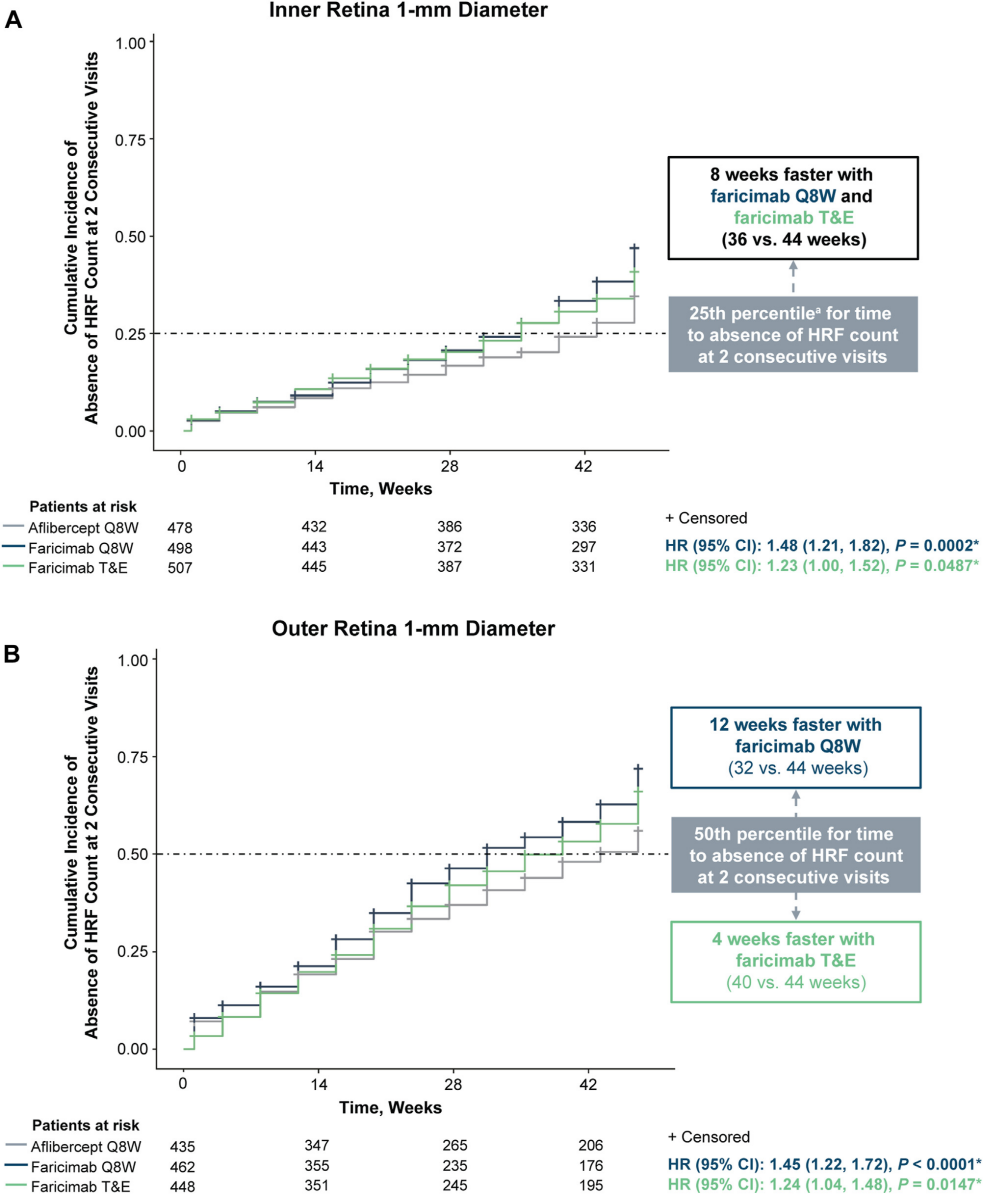
Cumulative incidence of absence of HRF count at 2 consecutive visits. ∗Nominal *P* value vs. aflibercept Q8W. Absence of HRF count was defined as the first time of HRF count = 0 for 2 consecutive visits. Patients who had such an event on or before the baseline visit were excluded from the corresponding analysis. Summaries of time to absence of HRF count are Kaplan–Meier estimates. Times represent the planned visit day. Results from stratified analyses are presented for HR and log-rank test vs. aflibercept. An HR >1 favors faricimab over aflibercept. ^a^The 50th percentile was not reached for inner retina 1-mm diameter across treatment arms. CI = confidence interval; HR = hazard ratio; HRF = hyperreflective foci; Q8W = every 8 weeks; T&E = treat-and-extend.

Time to absence of HRF count at 2 consecutive visits was achieved faster with faricimab Q8W and T&E compared with aflibercept Q8W in both the inner and outer retina, 1-mm diameter (Fig 7). In the inner retina, the 25th percentile was reached at 36 weeks with faricimab Q8W and faricimab T&E compared with 44 weeks with aflibercept Q8W (Fig 7A). Over 48 weeks, the hazard ratio for achieving absence of HRF count at 2 consecutive visits was 1.48 (95% confidence interval [CI] 1.21, 1.82; nominal *P* < 0.001) with faricimab Q8W and 1.23 (95% CI 1.00, 1.52; nominal *P* = 0.049) with faricimab T&E versus aflibercept Q8W (Fig 7A). In the outer retina, the 50th percentile was reached at 32 weeks with faricimab Q8W and 40 weeks with faricimab T&E compared with 44 weeks with aflibercept Q8W (Fig 7B). Over 48 weeks, the hazard ratio for achieving absence of HRF count at 2 consecutive visits was 1.45 (95% CI 1.22, 1.72; nominal *P* < 0.001) with faricimab Q8W and 1.24 (95% CI 1.04, 1.48; nominal *P* = 0.015) with faricimab T&E versus aflibercept Q8W (Fig 7B). Time to absence of HRF count at 2 consecutive visits was also assessed in both the inner and outer retina, 3-mm diameter. In both cases, the 25th percentile was not reached within 48 weeks with faricimab Q8W, faricimab T&E, or aflibercept Q8W (data not shown).

### Correlation between Change in HRF Volume and Change in IRF Volume at Week 48

Patients with greater decreases in HRF volume had greater reductions in IRF at week 48 in the total retina, 3-mm diameter (*r* = 0.42, *P* < 0.001; Fig S8, available at www.ophthalmologyscience.org).

## Discussion

In this post hoc analysis, we analyzed the substantial SD-OCT dataset from the phase III YOSEMITE/RHINE trials and showed that HRF volumes and counts decreased more with faricimab (regardless of regimen: Q8W or T&E) than with aflibercept. The difference was noticeable as early as week 16 (the completion of the head-to-head dosing period) and was maintained up to week 48 (the last study visit assessed in the current analysis) and was observed in the central 1-mm and 3-mm diameters of the retina, as well as the inner and outer retina. Hyperreflective foci volume and count decreased concurrently, showing that these foci did not simply get smaller but were also reducing in number, indicating resolution. Furthermore, we observed a faster time to absence of HRF count at 2 consecutive visits over 48 weeks in faricimab-treated versus aflibercept-treated patients within the 1-mm diameter for both the inner and outer retina.

The greater HRF decreases with faricimab versus aflibercept are likely a result of better DME disease control provided by dual Ang-2/VEGF-A inhibition over VEGF inhibition alone.^30^ In a post hoc analysis of YOSEMITE/RHINE, higher HRF volumes were seen with increasing CST, cystoid IRF, and Diabetic Retinopathy Severity Scale at baseline, suggesting that HRF are markers for DME disease severity.^22^ In support of this finding, greater decreases in HRF volume were associated with greater decreases in IRF volume at week 48 in the current study. Results from preclinical studies suggest that Ang-2 and VEGF-A synergistically regulate vascular leakage, inflammation, and neovascularization.^14^^,^^15^^,^^17^^,^^18^ In a recently published study evaluating vessel destabilization and inflammation in JR5558 mice, Ang-2 and dual Ang-2/VEGF-A inhibition were superior to VEGF-A inhibition alone in sustained (5 weeks) reduction of neovascularization and neovascular leakage.^14^ Similarly, only Ang-2 and dual inhibition, but not VEGF-A inhibition alone, reduced immune cell accumulation (CD11b+ [microglia/myeloid leukocytes], Iba1+ [microglia/macrophages], and CD45+ [leukocytes] cells) 5 weeks after treatment. Using a retinal ischemia-reperfusion injury model, the authors also demonstrated that dual Ang-2/VEGF-A inhibition was significantly better at preventing vascular permeability and neurodegeneration compared with blocking either Ang-2 or VEGF-A alone. In the primary year 1 and year 2 results of the YOSEMITE/RHINE trials, better anatomic outcomes were observed with faricimab compared with aflibercept and were achieved despite extended dosing in the faricimab T&E arm.^20^^,^^21^ In the present analysis, our findings of greater HRF reductions in faricimab-treated versus aflibercept-treated patients using robust methodology provide a potential biological explanation for the added benefit of Ang-2 inhibition in achieving more rapid and effective DME disease control. Similar findings have been observed for other DME biomarkers in patients treated with faricimab compared with aflibercept, including greater macular leakage resolution, faster time to first CST <325 μm and first absence of IRF, and reduced risk of epiretinal membranes.^30^ This body of evidence based on multiple biomarkers of DME severity is supported by the HRF results observed here, suggesting a clinically important role for dual Ang-2/VEGF-A inhibition with faricimab for the treatment of DME.

In the immediate period after treatment initiation, we observed that HRF volumes and counts increased in the inner retina, peaking at approximately week 8. A possible explanation for this finding is that some HRF may have been masked by the presence of diffuse edema and turbid/reflective/hazy IRF cysts at baseline, thus preventing detection by the algorithm. Turbid fluid in the retina is not uncommon in DME, manifesting as foci of varying contrast, and could mask the identification of small bright objects, causing an artificial rise in HRF volume.^31^^,^^32^ The HRF increase early in treatment may also be because of the precipitation of lipid/protein as the fluid is dried by treatment. In all 3 study arms of YOSEMITE and RHINE, CST reduced rapidly in the first 8 to 12 weeks,^20^^,^^21^ aligning well with the inflection point at which HRF volumes and counts began to decrease. From this point in the trials, the reduction in fluid was likely sufficient to allow for more accurate quantification of HRF. No such initial rise in the curves was seen in the outer retina, consistent with the fact that IRF largely affects the inner retina.

In the current study, we observed that HRF volume reductions with aflibercept were predominantly localized to the outer retina, with minimal changes in the inner retina. In contrast, treatment with faricimab reduced HRF volume in both the inner and outer retina. This observation also supports the better efficacy of faricimab, as prior research has shown the predilection for HRF to localize more extensively to the inner compared with the outer retina in the 1-mm diameter, which also represents the region where function is critical.^22^ The treatment response of HRF within specific retinal layers has been reported in previous studies;^31^^,^^33^, ^34^, ^35^ however, there is no clear consensus. The majority of reports evaluating change in HRF number before and after treatment, including corticosteroids and anti-VEGF therapy, observed a decrease in HRF in both the inner and outer retina.^31^ Migration of HRF toward the outer retinal layers has been observed in patients with DME after anti-VEGF treatment.^34^ In patients with DME treated with ranibizumab pro re nata, Yoshitake et al observed that eyes with HRF in the outer retinal layers had greater visual acuity gains and reductions in CST from baseline at 12 months versus eyes without such foci and that HRF in the outer, but not the inner retinal layers were associated with visual acuity improvement at 12 months.^35^ It has been hypothesized that HRF in the inner retina represents activated microglia based on the findings that the expression of CD14, a proinflammatory cytokine expressed by microglia, monocytes, and macrophages, correlated with HRF in the inner retina.^33^ An explanation for the observations in our study could be that the pathogenic mechanisms of HRF accumulation in the outer retina reflect a failure of the retinal RPE barrier and that VEGF alone may be responsible for this breakdown. In this case, both faricimab and aflibercept would be expected to be capable of helping to restore the outer blood–retinal barrier (i.e., the RPE), resulting in resolution of HRF. In contrast, inner blood–retinal barrier breakdown may be driven by both VEGF and proinflammatory cytokines, and thus dual Ang-2/VEGF-A inhibition with faricimab may restore the inner blood–retinal barrier more effectively than VEGF inhibition alone, allowing for better resolution of HRF in the inner retina. Further analyses may provide additional information to better understand the different effects observed with faricimab versus aflibercept on HRF location in the retinal layers.

Our study has several limitations. First, this was a post hoc analysis; the *P* values were nominal and not adjusted for multiplicity; therefore, no formal statistical conclusions can be made. Second, our data are limited by the density of and the spacing between the B-scans of the OCT volume scan. As the spacing between B-scans was ∼62 μm, some features, such as HRF located within areas that were not sampled, will not have been captured. Third, the current algorithm cannot track individual objects in a full 3-dimensional segmentation; this would require denser B-scans to achieve better resolution, for example, isotropic sampling with swept source OCT. Visual acuity gains were similar between faricimab and aflibercept through 2 years of the YOSEMITE/RHINE trials; future analyses will help determine the full clinical role of biomarker benefits associated with faricimab versus aflibercept, including HRF. Despite these limitations, our study provides the first comprehensive assessment of HRF in DME using a deep learning–based algorithm applied to images acquired within large, controlled clinical trials.

In conclusion, we have shown that dual Ang-2/VEGF-A inhibition with faricimab results in greater resolution of HRF, an important prognostic marker in DME, compared with VEGF inhibition alone with aflibercept; this difference was established at the end of the head-to-head dosing period (week 16) and maintained to week 48 for both the inner and outer retina. These data support the potential of faricimab to offer greater disease control for patients with DME.^20^^,^^21^ Our findings provide a foundation for future analyses that are needed to examine the functional relevance of HRF reduction using 2-year data from YOSEMITE and RHINE, and for other work, which will no doubt follow and help confirm a clinical role for this biomarker.

## Roche Data Sharing Statement

For eligible studies, qualified researchers may request access to individual patient-level clinical data through a data request platform. At the time of writing this request, the platform is Vivli. https://vivli.org/ourmember/roche/. For up-to-date details on Roche's Global Policy on the Sharing of Clinical Information and how to request access to related clinical study documents, see here: https://go.roche.com/data_sharing. Anonymized records for individual patients across more than 1 data source external to Roche cannot, and should not, be linked due to a potential increase in risk of patient reidentification.

## Supplementary data

Table S3

Clip S1
