# Supplementary material for: Effect of Faricimab versus Aflibercept on Hyperreflective Foci in Patients with Diabetic Macular Edema from the YOSEMITE/RHINE Trials
Source: Ophthalmol Sci. 2025 Apr 19;5(5):100798. doi: 10.1016/j.xops.2025.100798 (PMC12149427; doi:10.1016/j.xops.2025.100798)
Supplement: Figure S3 [file mmc1.pdf]

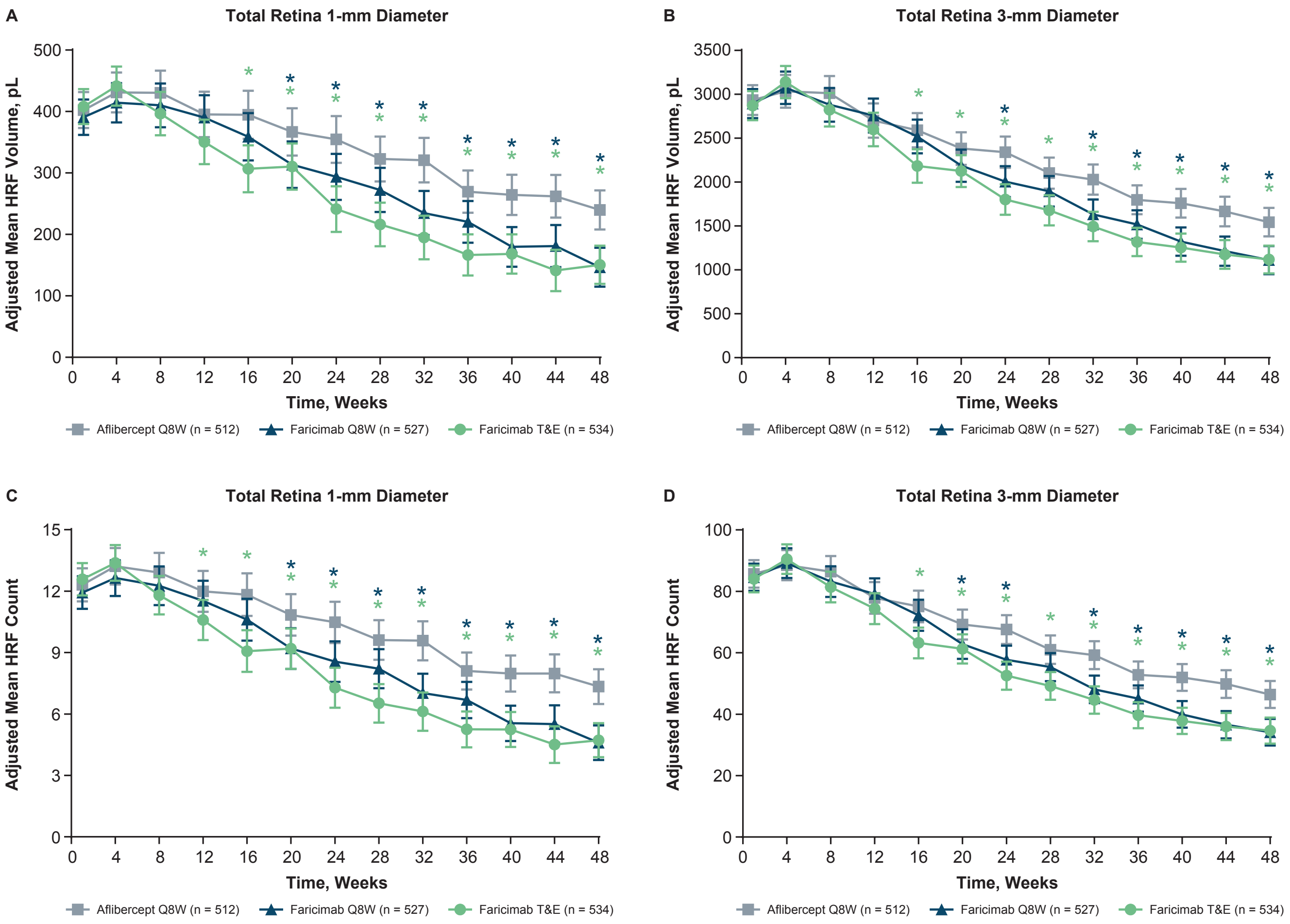

Figure S3. Analysis of hyperreflective foci (HRF) volumes and counts in the total retina for faricimab every 8 weeks (Q8W; blue), faricimab treat-and-extend (T&E; green), and aflibercept Q8W (gray) over time. HRF volumes in the total retina (A) 1 and (B) 3-mm–diameter Early Treatment Diabetic Retinopathy Study rings. HRF counts in the total retina (C) 1- and (D) 3-mm–diameter Early Treatment Diabetic Retinopathy Study rings. *Nominal $P < 0.05$ vs. aflibercept Q8W. Results and nominal P values were obtained using a mixed model for repeated measures analysis. Because the model is adjusted for baseline HRF value, no baseline values are shown in the figure. Error bars are 95% CI.

CI = confidence interval; pL = picoliters.
