# Supplementary material for: Effect of Faricimab versus Aflibercept on Hyperreflective Foci in Patients with Diabetic Macular Edema from the YOSEMITE/RHINE Trials
Source: Ophthalmol Sci. 2025 Apr 19;5(5):100798. doi: 10.1016/j.xops.2025.100798 (PMC12149427; doi:10.1016/j.xops.2025.100798)
Supplement: Figure S5 [file mmc2.pdf]

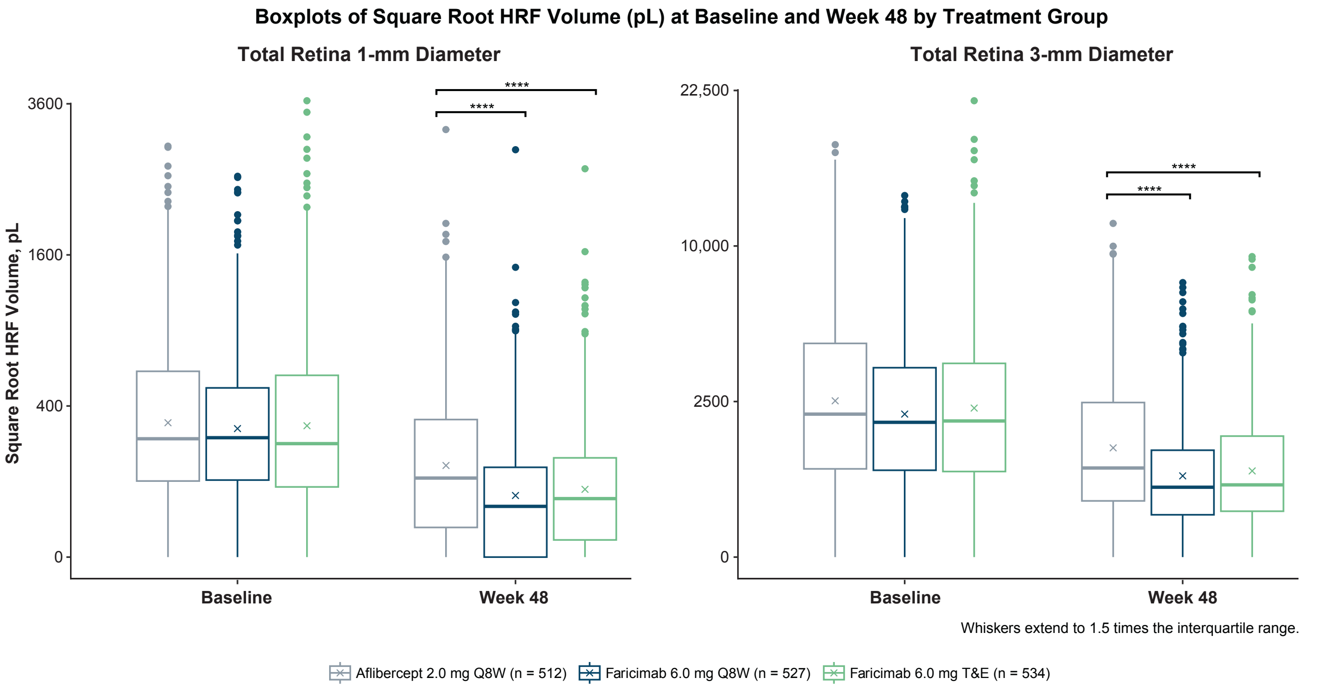

Figure S5. Baseline and week 48 measured hyperreflective foci (HRF) volumes in the total retina 1- and 3-mm–diameter Early Treatment Diabetic Retinopathy Study rings for faricimab every 8 weeks (Q8W; blue), faricimab treat-and-extend (T&E; green), and aflibercept Q8W (gray). Nominal *P* values, derived from the mixed model for repeated measures, are indicated as: ****$P \leq 0.0001$ vs. aflibercept every 8 weeks. Values are square-root transformed for improved visibility.
pL = picoliters.
