# Supplementary material for: Effect of Faricimab versus Aflibercept on Hyperreflective Foci in Patients with Diabetic Macular Edema from the YOSEMITE/RHINE Trials
Source: Ophthalmol Sci. 2025 Apr 19;5(5):100798. doi: 10.1016/j.xops.2025.100798 (PMC12149427; doi:10.1016/j.xops.2025.100798)
Supplement: Figure S8 [file mmc3.pdf]

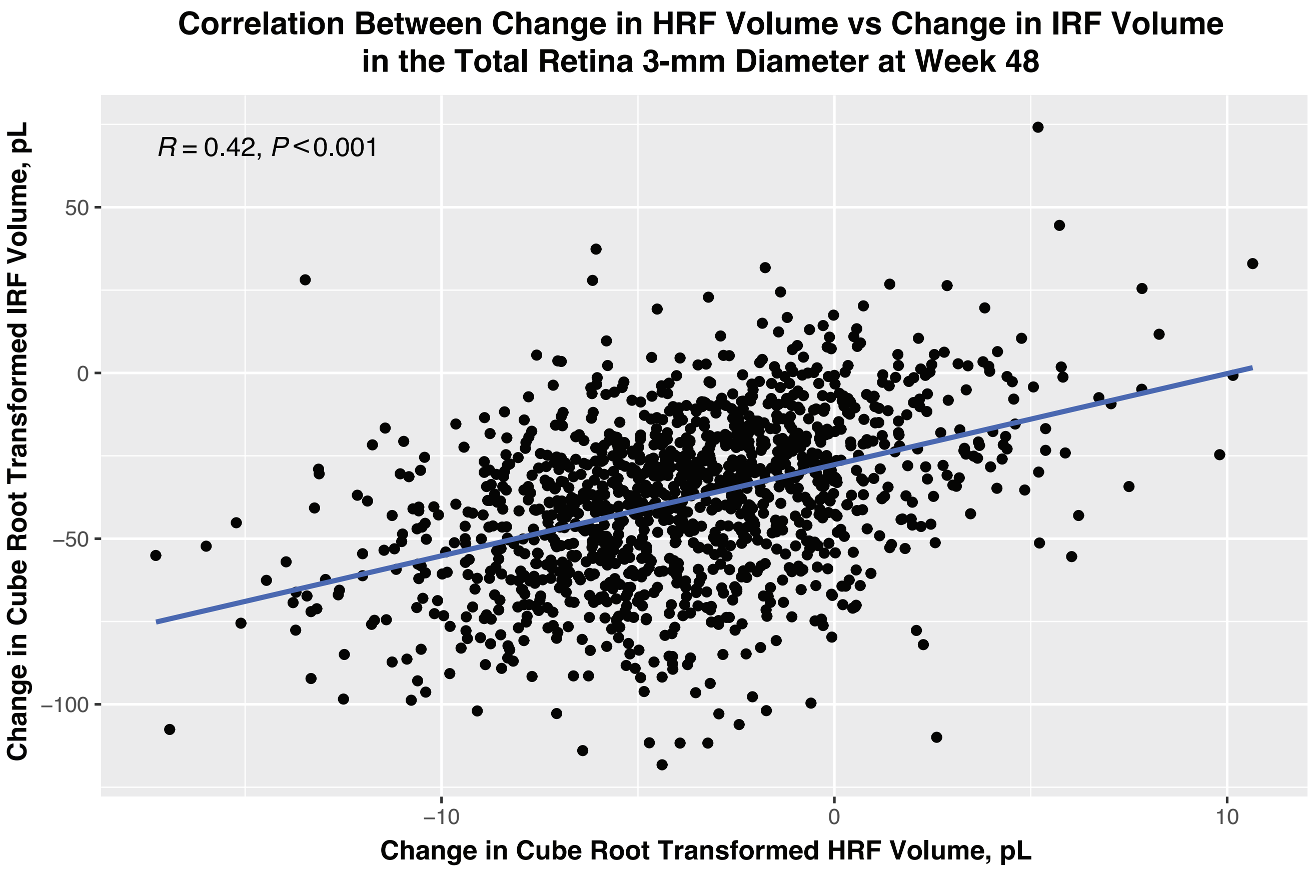

Figure S8. Correlation analysis between change in hyperreflective foci (HRF) volume and change in intraretinal (IRF) volume in the total retina 3-mm–diameter Early Treatment Diabetic Retinopathy Study ring at week 48. Blue line shows the linear regression. Pearson correlation coefficient and corresponding nominal *P* value are presented.
pL = picoliters
