# Supplementary material for: Effect of Faricimab versus Aflibercept on Hyperreflective Foci in Patients with Diabetic Macular Edema from the YOSEMITE/RHINE Trials
Source: Ophthalmol Sci. 2025 Apr 19;5(5):100798. doi: 10.1016/j.xops.2025.100798 (PMC12149427; doi:10.1016/j.xops.2025.100798)
Supplement: Table S2 [file mmc4.pdf]

Table S2. HRF Volumes at Baseline and Weeks 8, 16, and 48

	Faricimab 6.0 mg Q8W	Faricimab 6.0 mg T&E	Aflibercept 2.0 mg Q8W
**Inner retina, 1-mm**			
Baseline			
n	512	519	496
Mean volume (SE), pL	222.4 (11.9)	240.2 (14.5)	239.0 (14.0)
Median volume (IQR), pL	138.2 (40.5, 306.3)	121.8 (39.4, 308.1)	118.4 (38.7, 319.1)
Week 8			
n	456	460	439
Adjusted mean volume (SE), pL	273.5 (14.0)	272.1 (13.9)	289.3 (14.2)
Median volume (IQR), pL	173.2 (51.9, 361.5)	146.2 (39.0, 366.4)	177.7 (45.7, 381.2)
Week 16			
n	476	488	467
Adjusted mean volume (SE), pL	251.7 (15.7)	224.7 (15.5)	287.8 (15.9)
Median volume (IQR), pL	138.9 (33.7, 323.6)	95.8 (23.4, 302.5)	152.2 (35.5, 352.9)
Week 48			
n	414	449	421
Adjusted mean volume (SE), pL	104.1 (13.2)	110.1 (12.9)	180.3 (13.3)
Median volume (IQR), pL	27.6 (0, 105.9)	36.1 (0, 121.4)	65.6 (0, 235.0)
Across all visits			
P value (MMRM)*	0.0006	< 0.0001	
P value (Wilcoxon)*	< 0.0001	< 0.0001	
**Inner retina, 3-mm**			
Baseline			
n	512	519	496
Mean volume (SE), pL	1170.7 (51.6)	1310.2 (61.5)	1428.3 (70.0)
Median volume (IQR), pL	784.2 (320.2, 1647.8)	844.6 (379.5, 1752.3)	891.4 (359.3, 2013.5)
Week 8			
n	456	460	439
Adjusted mean volume (SE), pL	1591.0 (62.6)	1628.3 (62.2)	1669.2 (63.7)
Median volume (IQR), pL	1098.0 (467.3, 2097.5)	1154.0 (388.9, 2344.1)	1251.0 (478.3, 2469.9)

	Faricimab 6.0 mg Q8W	Faricimab 6.0 mg T&E	Aflibercept 2.0 mg Q8W
Week 16			
n	477	489	467
Adjusted mean volume (SE), pL	1534.3 (64.5)	1414.1 (63.9)	1608.4 (65.5)
Median volume (IQR), pL	1010.5 (358.2, 1901.3)	986.5 (323.1, 2028.8)	1082.7 (417.9, 2334.6)
Week 48			
n	414	449	421
Adjusted mean volume (SE), pL	763.9 (53.1)	777.2 (52.2)	1030.6 (53.7)
Median volume (IQR), pL	353.6 (130.9, 880.3)	390.2 (135.6, 1028.9)	616.4 (215.8, 1545.1)
Across all visits			
P value (MMRM)*	0.0142	0.0034	
P value (Wilcoxon)*	< 0.0001	< 0.0001	
**Outer retina, 1-mm**			
Baseline			
n	512	519	496
Mean volume (SE), pL	162.5 (11.8)	185.7 (14.0)	202.0 (14.7)
Median volume (IQR), pL	63.3 (9.0, 186.4)	63.1 (5.1, 213.8)	75.7 (5.5, 234.1)
Week 8			
n	455	460	439
Adjusted mean volume (SE), pL	131.9 (9.8)	126.5 (9.8)	146.8 (10.0)
Median volume (IQR), pL	44.6 (0, 150.5)	43.6 (0, 162.1)	49.0 (4.7, 170.4)
Week 16			
n	476	488	467
Adjusted mean volume (SE), pL	102.1 (8.8)	83.0 (8.7)	114.0 (8.9)
Median volume (IQR), pL	26.2 (0, 118.6)	25.9 (0, 106.4)	28.7 (0, 138.4)
Week 48			
n	414	449	421
Adjusted mean volume (SE), pL	37.2 (6.4)	41.4 (6.3)	65.7 (6.5)
Median volume (IQR), pL	0 (0, 33.1)	0 (0, 42.1)	8.3 (0, 68.6)
Across all visits			
P value (MMRM)*	0.0349	0.0004	
P value (Wilcoxon)*	< 0.0001	< 0.0001	
**Outer retina, 3-mm**			
Baseline			
n	512	519	496

	Faricimab 6.0 mg Q8W	Faricimab 6.0 mg T&E	Aflibercept 2.0 mg Q8W
Mean volume (SE), pL	1494.8 (84.9)	1656.5 (97.6)	1823.6 (105.6)
Median volume (IQR), pL	742.2 (238.4, 1991.7)	780.2 (209.1, 2118.5)	847.4 (265.0, 2441.8)
Week 8			
n	456	460	439
Adjusted mean volume (SE), pL	1252.3 (64.7)	1176.2 (64.2)	1361.0 (65.8)
Median volume (IQR), pL	545.8 (172.8, 1529.3)	575.0 (133.0, 1483.8)	680.9 (177.8, 1948.4)
Week 16			
n	477	489	467
Adjusted mean volume (SE), pL	945.8 (56.8)	754.6 (56.3)	1003.4 (57.7)
Median volume (IQR), pL	326.9 (89.9, 1059.4)	325.0 (90.9, 973.5)	433.8 (110.6, 1456.5)
Week 48			
n	414	449	421
Adjusted mean volume (SE), pL	311.7 (42.6)	319.6 (41.9)	533.6 (43.0)
Median volume (IQR), pL	85.5 (26.1, 273.9)	107.0 (33.0, 292.5)	188.0 (37.7, 663.3)
Across all visits			
P value (MMRM)*	0.0052	< 0.0001	
P value (Wilcoxon)*	< 0.0001	< 0.0001	
**Total retina, 1-mm**			
Baseline			
n	512	519	496
Mean volume (SE), pL	384.9 (19.0)	425.8 (23.1)	440.9 (23.0)
Median volume (IQR), pL	249.6 (103.9, 501.6)	225.5 (86.2, 578.8)	245.3 (101.4, 604.7)
Week 8			
n	456	460	439
Adjusted mean volume (SE), pL	410.0 (18.1)	396.6 (18.0)	430.3 (18.4)
Median volume (IQR), pL	247.7 (89.1, 538.4)	265.2 (61.6, 557.2)	276.5 (93.2, 607.1)
Week 16			
n	476	488	467
Adjusted mean volume (SE), pL	359.0 (19.6)	306.8 (19.5)	394.7 (19.9)
Median volume (IQR), pL	190.8 (46.6, 459.4)	149.6 (39.3, 451.9)	226.7 (61.9, 531.4)
Week 48			
n	414	449	421
Adjusted mean volume (SE), pL	146.6 (16.1)	150.6 (15.8)	240.0 (16.2)
Median volume (IQR), pL	45.1 (0.0, 141.2)	59.9 (5.1, 172.5)	109.4 (15.4, 331.4)

	**Faricimab 6.0 mg Q8W**	**Faricimab 6.0 mg T&E**	**Aflibercept 2.0 mg Q8W**
Across all visits			
P value (MMRM)*	0.0037	< 0.0001	
P value (Wilcoxon)*	< 0.0001	< 0.0001	
**Total retina, 3-mm**			
Baseline			
n	512	519	496
Mean volume (SE), pL	2665.5 (114.7)	2966.7 (136.4)	3251.9 (148.1)
Median volume (IQR), pL	1876.7 (779.0, 3705.5)	1914.2 (756.5, 3877.5)	2111.6 (804.0, 4718.4)
Week 8			
n	456	460	439
Adjusted mean volume (SE), pL	2880.9 (97.8)	2824.0 (97.1)	3012.8 (99.5)
Median volume (IQR), pL	1891.6 (778.3, 3676.9)	1969.4 (625.1, 3907.5)	2163.8 (862.1, 4475.1)
Week 16			
n	477	489	467
Adjusted mean volume (SE), pL	2519.4 (98.1)	2184.3 (97.2)	2591.6 (99.5)
Median volume (IQR), pL	1489.7 (576.6, 3174.3)	1475.1 (517.8, 3333.3)	1819.2 (596.7, 4048.4)
Week 48			
n	414	449	421
Adjusted mean volume (SE), pL	1112.3 (81.8)	1118.6 (80.4)	1543.6 (82.6)
Median volume (IQR), pL	504.2 (184.8, 1179.7)	538.8 (217.1, 1512.6)	820.0 (326.2, 2468.2)
Across all visits			
P value (MMRM)*	0.0112	0.0001	
P value (Wilcoxon)*	< 0.0001	< 0.0001	

1-mm = 1-mm–diameter Early Treatment Diabetic Retinopathy Study ring; 3-mm: 3-mm–diameter Early Treatment Diabetic Retinopathy Study ring; HRF = hyperreflective foci; IQR = interquartile range; MMRM = mixed model for repeated measures; pL = picoliters; Q8W = every 8 weeks; SE = standard error; T&E = treat-and-extend. *Comparison to aflibercept arm. All *P* values are nominal.
