# Supplementary material for: Effect of Faricimab versus Aflibercept on Hyperreflective Foci in Patients with Diabetic Macular Edema from the YOSEMITE/RHINE Trials
Source: Ophthalmol Sci. 2025 Apr 19;5(5):100798. doi: 10.1016/j.xops.2025.100798 (PMC12149427; doi:10.1016/j.xops.2025.100798)
Supplement: Table S3 [file mmc5.pdf]

Table S3. HRF Volumes in the Inner Retina at Select Visits

Mean Volume (SE), pL	Faricimab 6.0 mg Q8W (N = 519)	Faricimab 6.0 mg T&E (N = 524)	Aflibercept 2.0 mg Q8W (N = 502)
Inner retina, 1-mm			
Baseline	222.4 (11.9)	240.2 (14.5)	239.0 (14.0)
Week 8*	273.5 (14.0)	272.1 (13.9)	289.3 (14.2)
Factor (week 8/baseline)	1.23	1.13	1.21
Inner retina, 3-mm			
Baseline	1170.7 (51.6)	1310.2 (61.5)	1428.3 (70.0)
Week 8*	1591.0 (62.6)	1628.3 (62.2)	1669.2 (63.7)
Factor (week 8/baseline)	1.36	1.24	1.17

*Week 8 values are reported as adjusted means. 1-mm = 1-mm–diameter Early Treatment Diabetic Retinopathy Study ring; 3-mm: 3-mm–diameter Early Treatment Diabetic Retinopathy Study ring; HRF = hyperreflective foci; pL = picoliters; Q8W = every 8 weeks; SE = standard error; T&E = treat-and-extend.
